# Determining Arsenic Distribution in Urban Soils: A Comparison with Nonurban Soils

**DOI:** 10.1100/tsw.2002.187

**Published:** 2002-05-22

**Authors:** Tait Chirenje, L.Q. Ma, E.J. Zillioux

**Affiliations:** Soil and Water Science Department, University of Florida, Gainesville, FL 32611-0290, USA; Florida Power and Light, Environmental Services Department, P.O. Box 14000, Juno Beach, FL 33408, USA

**Keywords:** background concentrations, spatial variability, urban soils, outliers

## Abstract

There are many challenges in the determination of arsenic background concentrations in soils. However, these challenges are magnified when those determinations are carried out on urban soils. Irrespective of this, it is important to correctly identify and understand the extent of pollution in order to provide efficient preventative, remedial actions and cost-effective management of contaminated areas. This review paper discusses the factors that make the determination of arsenic background concentrations in urban areas different from similar determinations in nonurban areas. It also proposes solutions, where applicable, that are based on experience in determining arsenic background concentrations in both urban and nonurban areas in Florida, and from other studies in the literature. Urban soils are considerably different from nonurban areas because they have significant human disturbance, making them more difficult to study. They are characterized by high spatial and temporal variability, compaction, and modified chemical and physical characteristics. These differences have to be addressed during site selection, sample collection, and statistical analyses when determining arsenic distribution.

## INTRODUCTION

Large quantities of trace elements, including arsenic, have been cumulatively dispersed into the environment as a result of the ever growing demand for products, such as pesticides, herbicides, alloys, etc., that contain these elements. Knowing the distribution of arsenic in soils is important for assessing the impact of various natural and anthropogenic activities on soil contamination.

Various researchers have looked at the distribution of different trace elements in both urban[1,2,3,4] and nonurban[5,6] areas. Arsenic concentrations vary widely in both undisturbed soils (in different ecosystems) and urban areas (Table 1). In nonurban areas, soil is generally considered to be a growth medium for plants and agricultural production. In urban areas, soil forms an integral part of the surroundings; changes in its chemical nature can potentially expose large populations of people, including young children, to toxic materials[7]. Therefore, understanding the distribution of trace elements and their interactions with soils in urban areas is of prime importance.

The most important difference between urban and nonurban soils is that the properties of nonurban soils are mostly influenced by natural processes, while urban soils are subjected to considerable anthropogenic disturbance, involving mixing and the use of fill material and amendments[8,9]. Therefore, the behavior of nonurban soils can be extrapolated (mostly) from understanding the extent of influence of pedogenic activities on soil development[9,10], while those of urban soils are better extrapolated based on land use and land use history[2,9].

Due to the diversity of land use in urban areas, there is extremely high spatial and temporal variability among urban soils. Other factors that are unique to urban soils include: (1) compaction leading to modified structure[11]; (2) presence of surface crust on bare soil that is usually water repellent[12,13]; (3) modified pH; (4) restricted aeration and water drainage[14]; (5) interrupted nutrient cycling; (6) modified soil microbial activity; and (7) modified temperature regimes[9].

**TABLE 1**

**General Ranges of Arsenic Concentrations in Different Types of Soil**

| Soil Type | Range (mg/kg) |
|---|---|
| Natural undisturbed soils* | |
| Alluvial soils | 2.1–22.0 |
| Chernozems and dark prairie soils | 1.9–23.0 |
| Clay and clay-loamy soils | 1.7–27.0 |
| Forest soils | 1.5–16.0 |
| Light desert soils | 1.2–18.0 |
| Light loamy soils | 0.4–31.0 |
| Loess & soils on silt deposits | 1.9–16.0 |
| Organic light (or rich) soils | 0.1–48.0 |
| Silty prairie soils | 2.0–12.0 |
| Soils on glacial till and drift | 2.1–12.0 |
| Soils over granites and gneisses | 0.7–15.0 |
| Soils over limestones/calcareous rocks | 1.5–21.0 |
| Soils over volcanic rocks (or ash) | 1.0–93.2 |
| | |
| Urban soils | |
| Gainesville, Florida† | 0.22–660 |
| Miami, Florida† | 0.22–110 |
| Adelaide, Australia‡ | 0.2–16.0 |
| Sydney, Australia‡ | 0.60–11.0 |
| Ontario, Canada‡‡ | 0.50–47.0 |
| Denver, Colorado†† | 1.1– > 1,000 |

*Note:* Data from the following references: †[2], ††[3], *[49], ‡[50], and ‡‡[51].

Due to the aforementioned differences, concentrations of arsenic determined in urban areas do not necessarily reflect accurate background concentrations because they may not represent the true natural arsenic concentrations in soils before the influence of human activity. *Background concentration* is defined as “the inherent concentration of an element in a natural medium, e.g., parent material, soil, or sediments, excluding any anthropogenic additions”[14]. The term *baseline concentration* is more appropriate because it does not imply that the concentrations observed are natural arsenic concentrations[15]. Baseline concentration refers to the current prevailing concentration, which can be used as a yardstick against which future changes can be measured[2]. In fact, the term *arsenic distribution* is used in this paper as a general term in order to avoid implicating possible sources. Several researchers have tried partitioning the sources of contamination in different compartments of the environment with varying degrees of success[10,15,16,17]. Understanding these distributions can serve as a means to monitor the long-term effects of environmental pollution, especially in urban areas where human activities are constantly adding increasing amounts of trace elements to the environment.

This paper addresses the challenges faced in determining the distributions of arsenic in urban areas and some of the factors that need to be considered in carrying out such studies. Although these factors generally affect the determination of distributions for other elements (e.g., lead and cadmium) as well, this publication will concentrate on the factors that make arsenic determination different from other elements. It must be stressed that urban areas vary greatly amongst themselves. The sources of pollution depend on specific industrial, geological, climatic, and sociological conditions. The extent of influence of each factor varies for each city, depending on location, size, and extent of urbanization (development). Temporal variation also plays a significant role in urban areas due to the rapid changes in land use. Experience was drawn from the studies conducted by the authors in both urban (Daytona Beach, Fort Lauderdale, Gainesville, and Miami, all in Florida) and nonurban areas (state of Florida “natural” background study), and from studies by other researchers. Solutions on how to address the complex issues faced in determining arsenic distribution in urban areas are also discussed.

## SAMPLING CONSIDERATIONS

The sample selection criteria used in any study depends on the specific objectives of that particular study. The factors that make sampling in urban areas unique include: increased and localized arsenic loading, high spatial and temporal variability of urban soils (within both land use categories and cities), modified chemical and physical properties, and accessibility to sampling areas.

### Widespread Use of Arsenic in the Urban Environment

Although arsenic occurs naturally in a variety of minerals (being the $20^{th}$ most abundant element in the earth’s crust), its widespread use in both urban and nonurban environments has led to elevated concentrations in the environment. Although the relative contributions of each specific use is subject to great debate, it is generally agreed that the use of chromated copper arsenate (CCA)–treated wood[18], arsenic-containing herbicides and pesticides, pigments, growth promoters for poultry and pigs[19], and land application of semitreated industrial waste[10] all add considerable amounts of arsenic to the environment. Other activities that have been shown to transfer arsenic from one component to another include mining, smelting, and fossil fuel combustion. Tables 2 and 3 show the global arsenic fluxes and concentrations in both the atmosphere and the pedosphere.

**TABLE 2**

**Global Atmospheric Arsenic Fluxes and Concentrations[a]**

| | As flux (tons $year^{-1}$) |
|---|---|
| Emission | |
| Cu-smelting | 12,800 |
| Coal combustion | 6,240 |
| Herbicide use | 3,440 |
| Pb and Zn smelting | 2,210 |
| Glass production | 467 |
| Wood preservatives | 150 |
| Waste incineration | 78 |
| Steel production | 60 |
| Deforestation | 1,920 |
| Flaming of grassland | 1,000 |
| Release from soil | 160–6,200 |
| Wood as fuel | 425 |
| Sum | 28,230–54,270 |
| Deposition | |
| Northern Hemisphere | 73,600 |
| Southern Hemisphere | 4,300 |
| | |
| Remote areas | 0.31 g $ha^{-1}$ $year^{-1}$ |
| Rural areas | 3–10 g $ha^{-1}$ $year^{-1}$ |
| Urban areas | 300 g $ha^{-1}$ $year^{-1}$ |

a After [52].

**TABLE 3**

**Global Atmospheric Arsenic Fluxes and Concentrations[a]**

| | **As flux (tons $year^{-1}$)** |
|---|---|
| Solid waste from metal fabrication | |
| Municipal sewage, organic waste | 110 |
| Fertilizers | 250 |
| Urban refuse | 280 |
| Logging, wood waste | 400 |
| Animal, agricultural waste | 1,700 |
| Atmospheric deposition | 5,800 |
| Coal ashes | 13,000 |
| Discarded products | 22,000 |

[a] After [52].

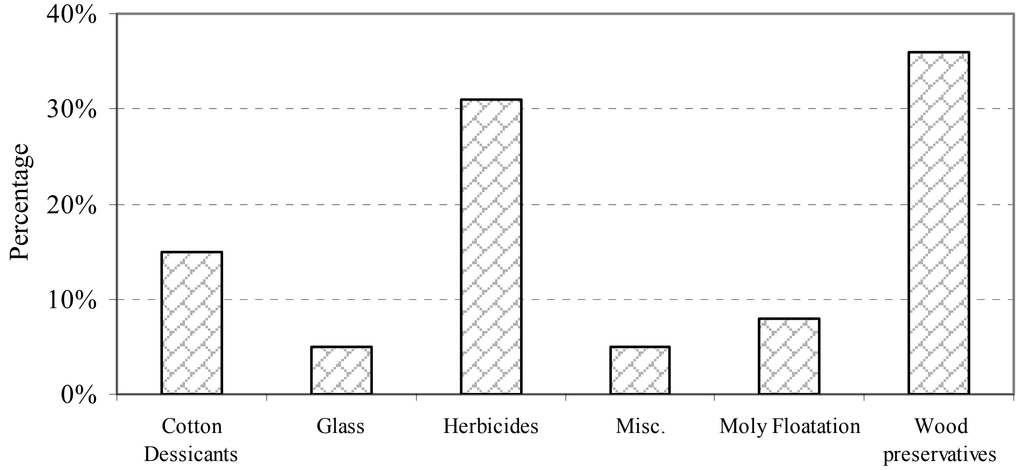

**FIGURE 1.** Estimated markets for arsenic trioxide in the United States in 1981[19].

The high efficiency of CCA-pressure treatment in reducing bacterial, fungal, and insect decay in wood[20] has led to the increased use of pressure-treated wood in warmer climates, where problems of accelerated wood decay are rampant. This has manifested itself in the proliferation of CCA-treated wood structures, such as CCA-treated wood in playground equipment, fences, and decks in most urban areas in the southern United States, including Florida[21]. There have been several cases of panic whenever elevated concentrations of arsenic have been detected, especially where there is potential risk to children.

Unlike agricultural areas, urban areas are characterized by zones of intense industrial activity (including smelting and fossil fuel burning plants), and these activities also lead to increased arsenic concentrations in localized areas. Applications of arsenic-containing herbicide and pesticide use in public utility right-of-ways also add arsenic to these areas. Contrary to the generally held misconception that agrochemicals are mostly used in nonurban areas, there is widespread use of fertilizers, herbicides, and pesticides to increase plant growth and control weeds, insects, and diseases in the landscaped sections of urban areas. The application of treated wastes in urban areas also contributes to nonpoint increase in trace element concentrations. Although application of treated wastes is generally considered safe, long-term application has been shown to increase loading of trace elements, including arsenic (which has an annual global loading of 0.01–0.24 metric tons from sewage sludge per year[7]).

Because the intensity of human impact depends on the land use in urban areas, it is prudent to use land use as a stratification criterion. However, the frequent change in land use in most urban areas further complicates this already complex situation. Consequently, studies carried out in urban areas not only have to take into consideration the potential of current land use to add arsenic to the environment, but they also have to consider historical land use. This can be a tedious and futile exercise depending on the quality of records maintained by the urban area of interest. This topic is discussed in more detail in a later section, entitled "Statistical Considerations."

It is important to differentiate those arsenic concentrations that may be a result of specific human activities (point sources) from those resulting from nonpoint sources in order to have a reliable baseline for establishing a reasonable standard for different land uses and soil conditions. This is important for those areas where cleanup may be required. For example, the Florida Department of Environmental Protection (FDEP) takes into account site-specific background concentrations of arsenic for sites that have arsenic concentrations above the soil clean-up target levels (SCTL) of 0.8 mg/kg[22,23,24]. Other states almost exclusively consider the site-specific background concentrations when regulating arsenic pollution[3,25].

A common dilemma faced in baseline studies lies in drawing boundaries between the areas to include and/or exclude during sampling. For example, is it necessary to exclude some areas that have activity patterns or land use patterns that increase arsenic concentrations although they form a large portion of the sampling area (e.g., heavy industry sites)? Most researchers ignore areas suspected of having been exposed to specific activities that add arsenic to the environment. Important distinctions have to be made depending on the objectives of each specific study.

## High Spatial Variability

The high spatial variability in urban areas has important implications for the number of samples that are collected for analyses. High spatial variability often leads to the requirement of larger sample sizes for statistically sound determinations at acceptable confidence levels. For example, many investigators use the following relationship to determine the number of samples needed for a given statistical level of significance[2,26]:

$$N = [(S \text{ x } t_{\alpha})/R]^2 \qquad (1)$$

where N is the number of samples needed, S is the estimated standard deviation of the mean concentration of previously collected samples in that area (preliminary study), $t_{\alpha}$ is the Student t value for a given confidence interval, and R is the accepted variability in mean estimation. The probability of making a Type I error, $\alpha$ (the error of concluding that a value is higher than a certain known threshold), is usually set at 0.05 (i.e., 1 chance in 20, hence the use of 95% confidence interval, [1-$\alpha$]*100%). This relationship depends on the spatial variability associated with the area to be studied. The higher the spatial variation, the larger the sample size required.

Alternatively, the following formula has been used:

$$n_0 = [\frac{z_{\alpha} + z_{\beta}}{D}]^2 + 0.5 * Z_{\alpha}{}^2 \qquad (2)$$

where *D* is the minimum detectable relative difference divided by the coefficient of variation, and $Z_{\alpha}$ and $Z_{\beta}$ are critical values from statistical tables (standard normal distribution[27]). For example, for 80% confidence and 95% power, $\alpha = 0.2$ and $\beta = 0.05$. Like Eq. 1, this formula is also dependent on the spatial variability of the area of interest.

However, it must be noted that these two methods are used when the objective of the determination focuses on the central tendency of the data. Most studies on undisturbed (nonurban) areas tend to focus on the mean and its related parameters. Methods that do not concentrate on the central tendency of the population exist[28]. For example, Conover[29] described a method to determine the number of samples needed to describe a given percentile of a distribution for a given level of confidence. This method allows investigators to compare data from different areas by using distribution relationships without concentrating on the mean or its related parameters. The main advantage of this approach is that less time is spent deciding the types of analyses (parametric or nonparametric methods), transformations (natural or log 10), and other design considerations while concentrating on distribution patterns.

Because urban soils vary widely within short distances, composite sampling, a popular sampling method in undisturbed areas, is highly undesirable in studies of urban areas. Composite sampling dilutes the effects of the lower and upper extremes collected at any site, conditions that are rare in nonurban areas. Although composite sampling reduces the number of samples analyzed as well as the cost (conditions that are convenient for undisturbed areas), discrete sampling is more appropriate in urban areas. For example, 12 composite samples consisting of 5 discrete samples each will have the same level of confidence in the mean as 60 discrete samples. However, the true variability of those 12 samples would be much lower than that of the 60 discrete samples. Discrete sampling is more appropriate in urban areas because it is more likely to pick out possibly contaminated sites in the highly variable sampling areas (possible outliers, depending on the objectives of the study).

## *Sampling Depth*

Another important difference between sampling in nonurban and urban soils is that the sampling depth in mostly agricultural soils is generally accepted as 15 cm (the depth of the plough layer) or A horizon for surface soils[6]. A consequence of vertical mixing of soil during development in urban soils is that it gives rise to vertical discontinuities. This renders the sampling schemes that usually work well for nonurban soils inapplicable. Pedogenic processes have led to well-understood horizonation in natural soils that can be separated visually by trained sample collectors. Such horizonation rarely exists in urban areas. In contrast, urban soils often have abrupt changes from one horizon to the next (lithological discontinuity) depending on the construction history of the site. This vertical variability (discontinuity) may lie between 6 and 35 cm below ground in street-side soils[11]. Each layer may be drastically different in texture, structure, organic matter content, pH, bulk density and related aeration, drainage, water holding capacity, and fertility. Some areas have more than one lithological discontinuity and this further complicates the selection of sampling depth to be used in assessing arsenic distribution in soils.

The Florida DEP uses the upper 60 cm of soil as the cut-off depth for surface soils. This depth represents the soil that humans are often exposed to and it includes the buried layer of soil that may be mixed with topsoil during construction and/or other activities. Various researchers have used different depths depending on their specific objectives. For instance, Brinkmann[30] collected samples from the top 3 cm of the profile within 1 m of the front entryway of homes at points located on a 1 km grid within the residential areas of the city of Tampa, FL, in a soil lead distribution study. Sample depths of 5 to 12 cm were used in a soil lead study in Minneapolis[5]; Davies et al.[31] collected samples from the top 5 cm of the profile in a lead study in Birmingham, England, while Chen et al.[1] and Wilcke et al.[32] collected samples from the top 5 cm of the profile in heavy metal monitoring studies in Hong Kong and Bangkok, Thailand, respectively.

Most researchers collect soil from the upper 5 or 10 cm of the profile because that layer represents the soil of greatest exposure to humans. A sampling depth of 60 cm, subdivided into three depths (0–20, 20–40, and 40–60 cm) was used in Gainesville, FL, to accommodate both the need to understand exposure at the surface and the distribution with depth to assess arsenic mobility[2]. These subsampling depths were adjusted to 0–10, 10–30, and 30–60 cm in a later study in Miami to reduce the increased dilution effects of the extra 10 cm of soil collected at subsurface (10–20 cm). It must be noted that this approach may yield misleading results if the sampled area has recently undergone disturbance, creating a buried layer while at the same time creating a fresh, nonimpacted surface soil.

## Modified Soil Properties

Increased human influence in urban areas leads to the introduction of foreign materials to soils, and a corresponding change in both chemical and physical characteristics. These have far-reaching influences on the vertical and lateral distribution of arsenic. For example, the change in chemical properties affects the reduction-oxidation states, as well as adsorption-desorption and volatilization-methylation reactions, while the changes in physical properties affect leaching, retention, and mobility.

### *Changes in Chemical Properties*

Conditions in urban areas — building construction, roads, mass communication and utility lines — have a marked impact on soil chemical properties. For example, among other things, the change in pH and other chemical properties can be attributed to (1) application of calcium or sodium chloride as road and sidewalk deicing compounds in northern latitudes, (2) irrigation of vegetation with calcium-enriched water, and (3) release of calcium from the weathering of building rubble comprised of masonry, cement, and plaster, and the surface weathering of sidewalks.

Most urban soils tend to have higher pH values than their natural counterparts[33]. Street-side soils of Syracuse, NY, have been shown to have pH values ranging from 6.6 to 9.0 (mean ~8.0), while soil pH in Philadelphia ranged from 3.7 to 9.0 (mean ~7.6). In Berlin, street-side soils had a pH of about 8, while forest soils a short distance from the street had pH values less than 4. In Gainesville, FL, the mean soil pH was 6.3, more than one pH unit greater than nondisturbed soils. Soil pH for Miami was greater than 7[34], which was comparable to nonurban soils in the surrounding areas.

Soil pH affects the solubility of soil organic matter, the chemical forms of Fe, Al, and Ca, soil variable charge, and the speciation of arsenic in soils, among other factors. The first three factors all have significant effect on arsenic adsorption and desorption, as well as its leaching, mobility, and transformations. The input of chemicals from weathering of buildings and pavements creates serious ion imbalances. Superimposed on this, urban soils do not have sufficient organic matter and aeration to allow for the extent of C, N, P, and S cycling that is common in natural ecosystems. Hence, microbial activity may be severely limited in some urban soils, leading to the absence of many soil invertebrates, notably earthworms. Nutrient cycling, normal in nonurban soils, may not occur to any significant extent in some urban soils, and this has a bearing on the temporal and spatial distribution of arsenic in these soils. For example, bacterial and fungal activity, essential for volatilization of arsenic, is considerably reduced in urban soils.

### *Changes in Physical Properties*

There are several factors unique to urban areas that affect soil physical properties. For example, increased foot and wheel traffic destroys vegetation and compacts the soil. Urban soil vegetation is also subject to damage and reduction of cover, leaving the soil bare and susceptible to crust formation and compaction. Bulk density values ranging from 1,740 to 2,180 $kg/m^3$ were observed in four profiles in the Mall in Washington, D.C.[12], while Craul and Klein[11] found values ranging from 1,540 to 1,900 $kg/m^3$ with a mean of 1,820 $kg/m^3$. Bulk densities above 1,700 $kg/m^3$ are detrimental to proper root penetration, reducing plant viability. Furthermore, the absence of the binding effect of roots and organic matter, a consequence of reduced vegetation, exacerbates the situation.

The direct impact of raindrops on the unprotected surface in urban areas disintegrates aggregates and washes the finer particles downwards where they clog pores[35]. Water movement and gaseous diffusion are also confined by piping, curbing, foundation walls, and subway and parking garage ceilings, all typical structures in urban areas. Water and air infiltration are therefore greatly reduced. Superimposed on this, fungal excretions, calcium and magnesium soaps common in urban soils, and the deposition of petroleum base aerosols and particulates on the soil surface (which change the contact angle of the water-solid interface) also lead to water repellency.

### *Introduction of Foreign Materials*

The modification of topography during urbanization creates man-made soil. This soil is typified by large proportions of man-made materials (e.g., masonry, wood and paper, glass, plastic, metal, asphalt, and organic wastes). These materials greatly change both the chemical and physical properties of the soil, creating conditions that are often not present in natural soils. Due to the high variability in urban soil use, the amounts and nature of introduced foreign materials vary spatially and temporally both within and between urban areas. Although natural undisturbed soils vary from area to area, the pedogenic processes that govern them are well understood; hence, the arsenic distribution patterns can be interpreted within the context of the processes that impact specific soil profiles. Urban soils are not understood to the same level of depth as undisturbed soils. This makes it more difficult to take information obtained from studies conducted in one area and extrapolate it to another.

### Accessibility of Sampling Areas

Most properties in urban areas are privately owned. This means that even though some areas in urban settings may fit all the appropriate sample selection criteria of the study, the ability to procure samples still depends on the willingness of proprietors of the affected properties to have their properties surveyed. Previous experience in Gainesville, FL, showed that more than half of the private property owners generally decline to have their properties sampled[2]. As a result, the authors compared samples collected from private properties with those collected close to the same private properties, but on the public utility right-of-way, to determine if these can be used as viable substitutes. Duplicate samples were also taken from soil under different land uses to determine the reproducibility of such sampling schemes.

The results of these studies showed that public utility right-of-way samples were equally representative of the area of interest as yard or commercial property samples[2]. The results from the duplicate analyses showed high variation between samples collected from locations within the same proximity (10 to 60% relative percent difference between samples, data not shown). This further supports the contention by many researchers that it is more important to concentrate on understanding the distribution characteristics of arsenic within each category than to focus on the central tendency of arsenic variation within each category. However, it must be stressed that the results from the duplicate study may be urban area–specific due to the high variability among urban areas.

## STATISTICAL CONSIDERATIONS

Urban areas possess characteristics different from those of nonurban areas. These differences manifest themselves in higher specialization in land use and higher exposure levels of soils to trace element contamination in urban areas. Therefore, there is higher complexity in the randomization processes and treatment of data from urban soils than in nonurban soils. Specifically, stratification and higher-end censoring play a more prominent role in urban studies than in nonurban areas.

## Stratification

A good sampling design in environmental studies must be simple, cost effective, and efficient enough to meet the needs of the study of interest. The sampling method is the most critical factor in determining the outcome of any study. A comparison of three sampling methods — regular, cellular stratified, and random — showed that the outcome of most studies is dependent on the sampling method[36].

An important difference between the sample populations in urban areas and those of nonurban areas is that the latter does not need stratification (blocking into different land use classes). Differences in levels of activity in land use categories (commercial, residential, and public land) in urban areas make it impossible to treat them as being internally homogeneous. Hence, land use categories form the basis for stratification in such areas[2,10]. Stratified random sampling has been applied successfully in different studies[37,38,39].

Stratified random sampling requires more complex analytical solutions. The results from each category are analyzed separately and then combined to form a single population[28]. The level of sophistication in the second step depends on whether an equal number of factors is used in each category (balanced versus unbalanced design) or if there are different replications, in which case weighting is recommended. Comparing distributions from drastically different categories can also present several challenges. Studies carried out in Miami and Gainesville, FL, have shown that, although land use categories were generally different, the magnitude of the difference was not very high and the relationship between land use categories was urban area–specific. It is highly recommended that investigators carefully consider the objectives of their specific study and have a good understanding of the area that they are working in before choosing a randomization and sampling scheme.

## Censoring

The presence of non-detects and suspiciously high concentrations presents one of the most difficult problems in the analysis of environmental monitoring data. A few excellent reviews on this subject exist[28]. The inclusion of values that are below the detection limit in the analyses produces data that have varying levels of certainty, hence violating the assumption of homoscedasticity (constant measurement variation). Several methods of dealing with censored data exist[40]. The choice of method depends on the degree of censoring (e.g., 10 versus 60% censoring), the type of application (e.g., computing the mean versus computing a prediction limit from data that are a mixture of quantifiable and nonquantifiable measurements), and ease of use[28]. There is considerable controversy over which methods to use because, depending on their inclination or bias, researchers can easily affect the outcome by choosing methods that are favorable to a particular end result.

Several methods of censoring high-end values (outliers) also exist[28,41,42]. Outliers are those values that do not conform to the pattern established by other observations. Outliers can arise from errors in transcription or data coding, analytical instrument failure, calibration errors, or underestimating the inherent spatial or temporal variability in constituent concentrations. It is important to exclude outliers in data computations because their inclusion increases the false negative rate, causing potentially contaminated measurements to be accepted as consistent with normal data.

A common method used with environmental data is to perform a log-transformation on the data, normalizing and plotting quantile-quantile (QQ) plots. This method relies on both visual and mathematical dexterity. A variation of this method is discussed in detail in Gilbert[41]. Other popular methods include Rosner's test[43], Kurtosis, Skewness, and the Shapiro-Wilks test[28]. The Shapiro-Wilks test is the most popular of these methods because of its simplicity. High-end censoring is not necessary if the objective is to determine data distributions[42]. Outliers have a tremendous impact on the mean, confidence intervals, and other related statistics.

## Normalization

Normalization of trace element concentrations with a soil factor (clay content and organic carbon) has been suggested[44]. This normalization compensates for the differences in retention and reaction of trace elements with soil constituents. However, because trace element variability is not only controlled by the grain size distribution or carbon content, but also by the parent material from which the soil is derived, several workers have proposed the use of conservative elements, such as Al[45], Li[46], Cs[47], and Fe[48] for normalization. A *conservative element* is a natural element that is structurally combined with one or more of the major trace element carriers in minerals[15].

Normalization has been used with varying degrees of success in rural and generally undisturbed soils. Table 4 shows the correlationships of arsenic to some of the soil properties commonly used in normalization. However, normalization is not applicable to urban areas because of the added complications brought on by the different land use categories and the widespread use of fill. In fact, Short et al.[33] described the parent material of soils in the Mall area of Washington, D.C., as locally derived miscellaneous fill. Therefore, the same information that is usually easily discernible from determinations in nonurban soils — vertical and lateral distribution patterns with respect to time and natural processes — is not available from urban soils.

**TABLE 4**

**Correlation Coefficients of Arsenic Concentrations with Soil Properties in Urban and Nonurban Areas[a]**

| Element | pH[†] | Clay[††] | OC[‡] | TOTAL FE[‡‡] | TOTAL AL[‡‡] |
|---|---|---|---|---|---|
| Florida soils | | | | | |
| Nonurban (n = 448) | 0.14 | 0.33 | 0.58 | 0.66 | 0.60 |
| Gainesville (n = 201) | 0.10 | 0.01 | -0.05 | -0.04 | 0.02 |
| Miami (n = 260) | 0.09 | 0.04 | -0.08 | -0.09 | -0.06 |
| Other soils | | | | | |
| Nonurban (n = 222) | 0.76 | 0.93 | -0.01 | 0.99 | — |

[a] After [34].
[†] pH is determined in water (soil:water ratio 1:5) using an Accumet Model 20 pH meter.
[††] Clay particle size analyses were determined by the pipette method.
[‡] OC determined by the Walkley-Black method.
[‡‡] Total Fe and Al were determined on an inductively coupled Plasma (ICP-spectrophotometer) after extraction with $HNO_3$.

## CONCLUSIONS

This paper looked at the challenges faced in determining arsenic distribution in urban soils. Several factors that distinguish urban soils from nonurban soils were discussed. The most important factor was that humans are the most dominant geomorphic agent in the development of urban soils, while natural factors, climate, time, biological activity, topography, and soil parent material predominate in natural soils. Therefore, arsenic distribution in urban areas is affected both by land use and by a plethora of processes that are unique to urban soils. These processes not only affect sample selection and randomization, but also data analyses. For example, data from urban areas is analyzed using land use as a criterion for blocking. The high variability of urban soil samples within land use class and within the area of interest affects the size and nature of samples collected. It is very difficult to come to a unified approach in dealing with urban soils due to their high variability and the immense differences among urban areas. Nonetheless, reliable, reproducible results can be obtained if the factors discussed in this paper are taken into consideration.
